# Uterine fibroid management: from the present to the future

**DOI:** 10.1093/humupd/dmw023

**Published:** 2016-10-20

**Authors:** Jacques Donnez, Marie-Madeleine Dolmans

**Affiliations:** 1 Professor EM, Catholic University of Louvain, Director, Société de Recherche pour l'Infertilité (SRI), 143 Avenue Grandchamp, B-1150 Brussels, Belgium; 2 Gynecology Department, Cliniques Universitaires St-Luc, Avenue Hippocrate 10, 1200 Brussels, Belgium; Pôle de Gynécologie, Institut de Recherche Expérimentale et Clinique, Université Catholique de Louvain, Avenue Mounier 52, bte B1.52.02, 1200 Brussels, Belgium

**Keywords:** uterine fibroids, leiomyomas, selective progesterone receptor modulators, ulipristal acetate, surgery, medical therapy, myomectomy

## Abstract

Uterine fibroids (also known as leiomyomas or myomas) are the most common form of benign uterine tumors. Clinical presentations include abnormal bleeding, pelvic masses, pelvic pain, infertility, bulk symptoms and obstetric complications.

Almost a third of women with leiomyomas will request treatment due to symptoms. Current management strategies mainly involve surgical interventions, but the choice of treatment is guided by patient's age and desire to preserve fertility or avoid ‘radical’ surgery such as hysterectomy. The management of uterine fibroids also depends on the number, size and location of the fibroids. Other surgical and non-surgical approaches include myomectomy by hysteroscopy, myomectomy by laparotomy or laparoscopy, uterine artery embolization and interventions performed under radiologic or ultrasound guidance to induce thermal ablation of the uterine fibroids.

There are only a few randomized trials comparing various therapies for fibroids. Further investigations are required as there is a lack of concrete evidence of effectiveness and areas of uncertainty surrounding correct management according to symptoms. The economic impact of uterine fibroid management is significant and it is imperative that new treatments be developed to provide alternatives to surgical intervention.

There is growing evidence of the crucial role of progesterone pathways in the pathophysiology of uterine fibroids due to the use of selective progesterone receptor modulators (SPRMs) such as ulipristal acetate (UPA). The efficacy of long-term intermittent use of UPA was recently demonstrated by randomized controlled studies.

The need for alternatives to surgical intervention is very real, especially for women seeking to preserve their fertility. These options now exist, with SPRMs which are proven to treat fibroid symptoms effectively. Gynecologists now have new tools in their armamentarium, opening up novel strategies for the management of uterine fibroids.

## Introduction

Uterine fibroids (also known as leiomyomas or myomas) are the most common form of benign uterine tumors (Stewart, 2001; Donnez and Jadoul, 2002; Bulun, 2013; Islam *et al*., 2013; Drayer and Catherino, 2015). They are monoclonal tumors of uterine smooth muscle, thus originating from the myometrium (Kim and Sefton, 2012; Bulun, 2013; Islam *et al*., 2013). They are composed of large amounts of extracellular matrix (ECM) containing collagen, fibronectin and proteoglycans (Parker, 2007; Sankaran and Manyonda, 2008; Kim and Safton, 2012). Leiomyomas occur in 50–60% of women, rising to 70% by the age of 50 (Baird *et al*., 2003), and, in 30% of cases, cause morbidity due to abnormal uterine bleeding (heavy menstrual bleeding inducing anemia) and pelvic pressure (urinary symptoms, constipation and tenesmus) (Donnez and Jadoul, 2002; Donnez *et al*., 2014a,b). Clinical presentations of uterine leiomyomas include pelvic masses, pelvic pain, infertility and obstetric complications (Donnez and Jadoul, 2002).

### Risk factors

**Figure 1:**
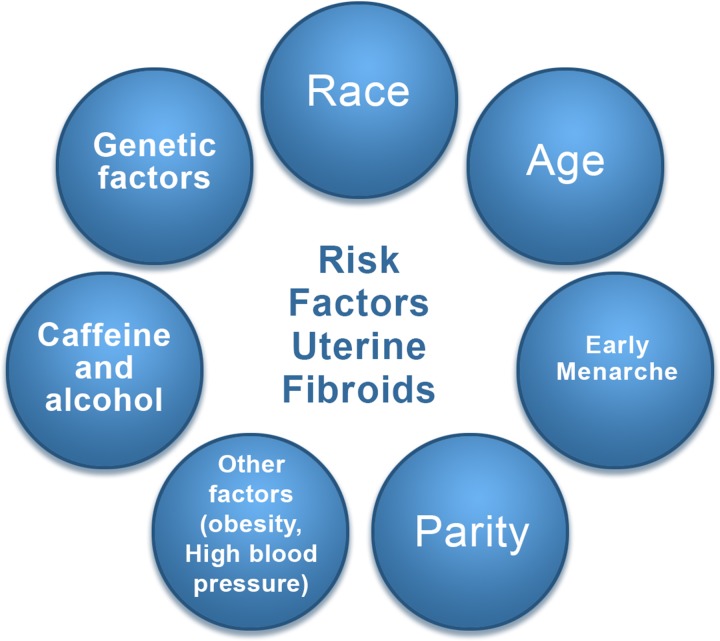
Risk factors for uterine fibroid. These include race, age, delayed pregnancy, early menarche, parity (protective effect), caffeine, genetic alterations, and others, such as obesity and a diet rich in red meat.

The risk factors for uterine fibroids are illustrated in Fig. 1.

#### Race

Race constitutes an important risk factor for leiomyoma development (Marshall *et al*., 1997; Wise *et al*., 2004; Stewart *et al*., 2013; El Toukhi *et al*., 2014). An US study found that the incidence of uterine fibroids was 60% by age 35 among African-American women, increasing to >80% by age 50, while Caucasian women showed a rate of 40% by age 35, increasing to 70% by age 50 (Baird *et al*., 2003). Differences in gene expression in uterine fibroids between these two groups may influence these growth rates (Davis *et al*., 2013). Nevertheless, it is clear that African-American women have a greater chance of being affected by uterine fibroids, particularly at an earlier age (Wise *et al*., 2004, 2005; Wise and Laughlin-Tommaso, 2016). Among women of African origin living in Europe, a similar trend has been observed, with more severe symptoms and surgery required at a younger age. Moreover, recurrence rates after surgery (myomectomy) may be as high as 59% after an interval of 4–5 years (Malone, 1969; Donnez *et al*., 2014a,b) for women of African origin.

#### Age

Peddada *et al*. (2008) followed the size of 262 leiomyomas from 72 women for up to 12 months using magnetic resonance imaging (MRI). The average growth rate was 9% over 6 months, but growth rates differed between races when age was taken into account. White women under 35 years of age had faster-growing tumors than white women over 45, who exhibited a comparatively slow growth rate. On the other hand, women of African origin did not show any decrease in myoma growth rates with age.

Moreover, delaying the first pregnancy until the third decade of life also places women at higher risk of uterine fibroids (Petraglia *et al*., 2013).

#### Early menarche

Menarche at an early age increases the risk of developing fibroids and is also considered a risk factor for other hormonally mediated diseases, such as endometrial and breast cancers (Kim and Sefton, 2012; Khan *et al*., 2014).

#### Parity

Pregnancy has been found to have a protective effect on the development of uterine fibroids, but the mechanism remains unclear. It has been suggested that during post-partum uterine remodeling, small lesions may be subject to selective apoptosis. Furthermore fibroid tissue may be highly susceptible to ischemia during both uterine remodeling and parturition (Baird and Dunson, 2003; Laughlin *et al*., 2010).

#### Caffeine and alcohol

An association has been reported between alcohol and caffeine intake and an increased risk of developing uterine fibroids in a study concerning the health of women of African origin (Wise *et al*., 2004; Wise and Laughlin-Tommaso, 2016).

#### Genetic factors

Some specific genetic alterations are linked to fibroid growth (Mäkinen *et al*., 2011; Eggert *et al*., 2012; Islam *et al*., 2013; Mittal *et al*., 2015; Styer and Rueda, 2015). Mehine *et al*. (2013) performed whole genome sequencing and gene expression profiling of 38 uterine leiomyomas and corresponding myometrium. The common occurrence of chromothripsis in uterine fibroids suggests that it also plays a role in their genesis and progression (Mehine *et al*., 2013, 2014).

#### Other factors

General health status may also be predictive of leiomyoma growth, with factors such as obesity and high blood pressure playing a role. A diet rich in red meat appears to increase the risk of developing leiomyomas, while smoking decreases the risk, for unknown reasons (Kim and Sefton, 2012; Islam *et al*., 2013).

### Classifications

Numerous classifications of myomas can be found in the literature (Lasmar *et al*., 2005; Stamatellos and Bontis, 2007). All of them take into account the degree of intramural extension and/or uterine cavity distortion. The fibroid classification adopted by the ESGE (European Society for Gynecological Endoscopy) has the advantage of being very simple (G0 is a pedunculated intrauterine myoma, G1 has its largest part (>50%) in the uterine cavity, and G2 has its largest part (>50%) in the myometrium).

**Figure 2:**
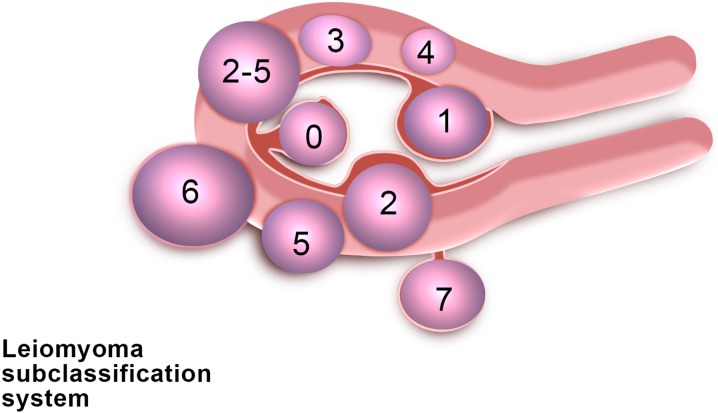
FIGO classification of uterine fibroids according to Munro *et al*. (2011). Fibroid types range from 0 to 8. 0 = Pedunculated, intracavitary; 1 = Submucosal, <50% intramural; 2 = Submucosal, ≥50% intramural; 3 = Contact with endometrium, 100% intramural; 4 = Intramural; 5 = Subserosal, ≥50% intramural; 6 = Subserosal, <50% intramural; 7 = Subserosal, pedunculated; 8 = Other (e.g. cervical, parasitic). Where two numbers are given (e.g. 2–5), the first number refers to the relationship with the endometrium, while the second number refers to the relationship with the serosa; e.g. 2–5 = Submucosal and subserosal, each with less than half the diameter in the endometrial and peritoneal cavities respectively. Fibroid classification cartoon republished with permission from Munro *et al*. (2011).

More recently, the FIGO classification was published (Munro *et al*., 2011), describing eight types of fibroids as well as a hybrid class (association of two types of myomas) (Fig. 2). As different types of fibroids are often present at the same time (depending on the site), this classification offers a more representative ‘map’ of fibroid distribution and will be used further for the establishment of new algorithms.

### Symptoms

Many fibroids are asymptomatic, but in 30–40% of cases, they show a variety of symptoms, depending on the location and size. Fibroids can cause heavy menstrual bleeding with subsequent anemia, which could be life-threatening (Parker, 2007; Nelson and Ritchie, 2015). African-American women have more severe symptoms in terms of heavy bleeding and anemia compared to white women (Stewart *et al*., 2013). Large fibroids can also result in pressure symptoms (bulk symptoms) that may be responsible for bowel and bladder dysfunction, including urgency, increased daytime urinary frequency and urinary incontinence (Gupta *et al*., 2008). Abdominal distention or distortion and pelvic pressure on the ureters (causing hydronephrosis) and pelvic blood vessels (particularly pelvic veins) could also interfere with quality of life (QoL) (Spies *et al*., 2002; Donnez *et al*., 2014a,b).

Dysmenorrhea and pelvic pain are frequently encountered, impacting on QoL and impairing daily activities (Spies *et al*., 2002). Infertility and recurrent miscarriage may also be symptoms of fibroids, depending on their location and size, especially for submucous and intramural myomas distorting the uterine cavity (Pritts *et al*., 2009; Sunkara *et al*., 2010, Yan *et al*., 2014; Zepiridis *et al*., 2015).

Fibroids can impair fertility through several possible mechanisms, including: (1) alteration of the local anatomy (anatomic distortion of the uterine cavity), with subsequent alterations to endometrial function (Somigliana *et al*., 2007); (2) functional changes, such as increased uterine contractility and impairment of the endometrial and myometrial blood supply (Donnez and Jadoul, 2002) and (3) changes to the local hormone milieu and paracrine molecular changes induced by fibroids, which could impair gamete transport and/or reduce blastocyst implantation (Sinclair *et al*., 2011; Galliano *et al*., 2015).

Moreover, fibroids can affect obstetric outcomes. Inflammatory pathways, associated or not with other reproductive disorders, may impair pregnancy outcomes (Vannuccini *et al*., 2016). Fibroids are significantly associated with preterm delivery (<37 weeks), primary cesarean section, breech presentation and lower birthweight infants (Shavell *et al*., 2012; Lam *et al*., 2014, Parazzini *et al*., 2015; Blitz *et al*., 2016). Very recently, a higher incidence of short cervix was also observed during pregnancy in women with fibroids (Blitz *et al*., 2016).

### Diagnosis

#### Pelvic examination

Examination of the pelvis may reveal an enlarged uterus or mass. If fibroids are suspected and a woman reports heavy menstrual bleeding, a hemoglobin evaluation will allow detection of iron deficiency anemia.

#### Ultrasonography

An ultrasound is the gold standard test for uterine fibroids. Its widespread availability enables easy and inexpensive confirmation in almost all instances. Moreover, ultrasonography after infusion of saline into the uterine cavity can delineate submucous myomas and indicate the proximity of intramural myomas to the endometrial cavity (Seshadri *et al*., 2015). The advent of 3D imaging technology has seen 3D ultrasound establishes itself as a useful tool for the investigation of myometrial pathology due to its ability to reconstruct the coronal plane of the uterus (Andreotti and Fleischer, 2014; Wong *et al*., 2015).

#### Hysteroscopy

A hysteroscopy may be required to differentiate intracavitary myomas and large endometrial polyps (Bettocchi *et al*., 2003; Di Spiezio Sardo *et al*., 2010; Parazzini *et al*., 2015). Hysteroscopy is usually performed in an outpatient setting and does not require any anesthesia (Bettocchi *et al*., 2003). Ultrasonography with saline infusion and diagnostic hysteroscopy should be considered more as complementary examinations when hysteroscopic myomectomy is indicated. Of course, in case of irregular bleeding or if the patient has risk factors for endometrial hyperplasia (obesity, chronic anovulation), hysteroscopy may be combined with an endometrial biopsy.

#### Magnetic Resonance Imaging

**Figure 3:**
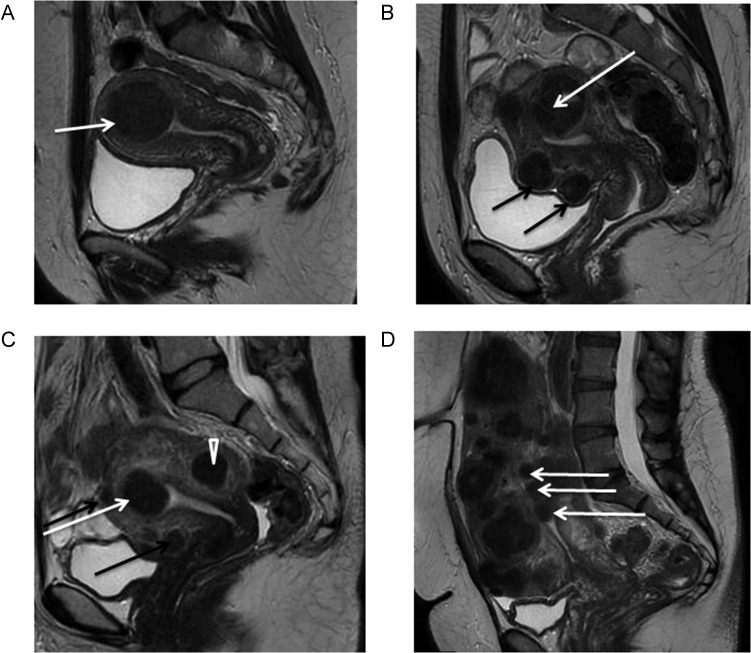
Magnetic resonance imaging (MRI) of fibroids. Midline sagittal T2-weighted images show different types of myomas according to the FIGO classification (Munro *et al*., 2011). Fibroids vary in size, number and site in the uterus. **(A)** Submucosal type 2 myoma. **(B)** Large type 2–5 myoma (white arrow): submucosal and subserosal, each with less than half the diameter in the endometrial and peritoneal cavities respectively. Subserosal type 5 myomas (subserosal, >50% intramural) (black arrows). **(C)** Submucosal type 2 myoma (>50% intramural) (white arrow). Intramural type 4 myoma (arrowhead). Small type 5 myomas (black arrows). **(D)** Multiple myomas, three of which are type 0 (intracavitary) (white arrows).

MRI can provide information on the number of fibroids, their size, vascularization, relationship with the endometrial cavity and serosal surface, and boundaries with normal myometrium (Fig. 3). It should nevertheless be stressed that like ultrasonography, MRI cannot diagnose malignancy with any certainty (Lumsden *et al*., 2015; Stewart, 2015). While MRI findings can suggest a diagnosis of sarcoma, there is currently no form of preoperative testing which can definitively rule it out (Lin *et al*., 2015). Possibly in the future, new types of imaging will improve the accuracy of detecting sarcoma, which remains a very infrequent condition (1/1500 in women aged <40 years and 1/1100 in women aged 40–44) (Wright *et al*., 2014).

## Current surgical management strategies

As stressed by Stewart (2015), there are areas of uncertainty surrounding the management of myomas, as only a few randomized trials have compared different therapies for fibroids. Moreover, data on their comparative effectiveness in terms of future fertility are lacking. There are also inadequate data on long-term outcomes in women who have undergone hysterectomy according to indication (Stewart, 2015). Prospective data and studies are essential to compare different options and evaluate long-term outcomes with regard to QoL, recurrence of symptoms (bleeding, bulk symptoms), fertility and even complications.

Indeed, in a cohort study of 30 117 Nurse's Health Study participants undergoing hysterectomy for benign disease, bilateral oophorectomy was found to be associated with increased mortality in patients under 50 years of age who had never used estrogen therapy (Parker *et al*., 2013).

While guidelines exist in the literature (ACOG, 2008; ASRM, 2008; Marret *et al*., 2012; Stewart, 2015), the risks and benefits of each option should be discussed with the patient. It should also be stressed that many other factors need to be taken into account, including the skill of the surgeons involved, as well as the experience of different centers in the available techniques.

**Figure 4:**
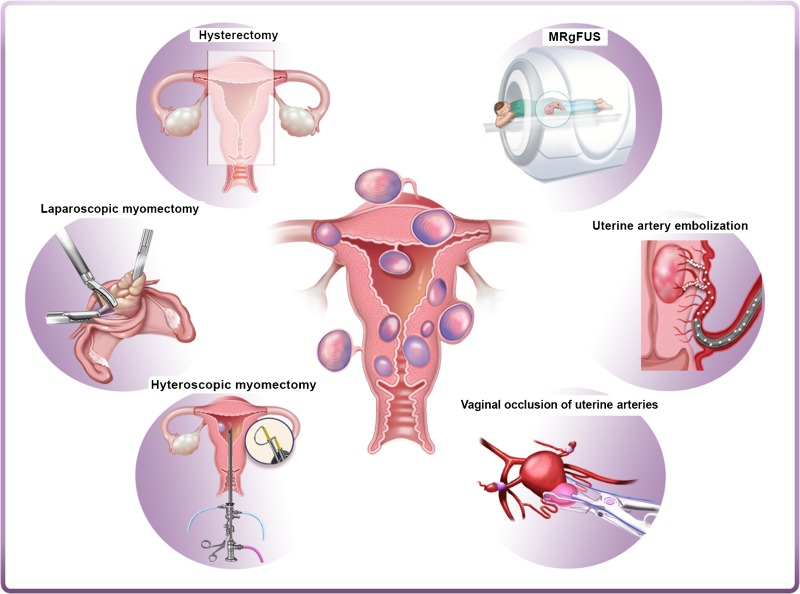
Current surgical and non-surgical management strategies of myomas. Left panel: hysterectomy, laparoscopic myomectomy and hysteroscopic myomectomy are the most widely used surgical interventions for myomas. Right panel: alternatives to surgical intervention include uterine artery embolization (UAE), high-frequency magnetic resonance-guided focused ultrasound surgery (MRgFUS) and vaginal occlusion of uterine arteries.

Current management strategies involve mainly surgical interventions, but the choice of treatment is guided by the patient's age and desired to preserve fertility or avoid ‘radical’ surgery such as hysterectomy (Donnez and Jadoul, 2002; Practice Committee of the American Society for Reproductive Medicine, 2008; Lumsden *et al*., 2015). Other surgical and non-surgical approaches include myomectomy by hysteroscopy, myomectomy by laparotomy or laparoscopy, uterine artery embolization (UAE) and other interventions performed under radiologic or ultrasound guidance (Fig. 4) (Donnez and Jadoul, 2002; Practice Committee of the American Society for Reproductive Medicine, 2008; Lumsden *et al*., 2015; Stewart, 2015; Zupi *et al*., 2015).

### Hysteroscopic myomectomy

Over the last 30 years, advances in instruments and techniques have promoted hysteroscopic myomectomy to the rank of a standard minimally invasive surgical procedure for submucous myomas. Small fibroids (<2 cm) are now routinely removed in an outpatient setting according to the technique described by Bettocchi (Bettocchi *et al*., 2003, 2004; Di Spiezio Sardo *et al*., 2010; Casadio *et al*., 2011; Mazzon *et al*., 2015; Vilos *et al*., 2015).

Depending on personal experience and available equipment, the gynecologist has a choice of several alternative procedures.

The first involves cutting the base of pedunculated fibroids with either the resectoscopic loop or laser fiber (Stamatellos and Bontis, 2007; Bettocchi *et al*., 2004; Di Spiezio Sardo *et al*., 2008; Tan and Lethaby, 2013). The base of the pedicle is cut and the fibroid is extracted by forceps or may be left in place.

The second alternative is a complete excision of fibroids by a one-step procedure (Di Spiezio Sardo *et al*., 2008, 2015). The most commonly used approach is the slicing technique. Repeated and progressive passage of the cutting loop allows the surgeon to cut the myoma into small chips. The operation is considered complete when the fasciculate fibers of the myometrium are visualized (Donnez *et al*., 1990; Bettocchi *et al*., 2004; Di Spiezio Sardo *et al*., 2015; Mazzon *et al*., 2015; Saridogan, 2016). Hysteroscopic resection is effective and safe and should be considered the technique of choice for type 1 myomas. The development of intrauterine morcellators has facilitated the implementation of hysteroscopic myomectomy (Lee and Matsuzono, 2016; Munro, 2016). If the myoma is large (>3 cm in diameter), there is an increased risk of operative complications (perforation, bleeding and fluid intravasation) and damage to surrounding myometrium due to use of electrosurgery. Interestingly, Casadio *et al*., (2011) demonstrated that during surgery, myometrial thickness increases when myoma slices are removed, leading to protrusion of the intramural component into the uterine cavity.

The third alternative is myomectomy by a two-step procedure (for large type 1–3 myomas of according to the FIGO classification, Munro *et al*., 2011). After resection or ablation of the protruded portion of the myoma during first-step hysteroscopy, the residual intramural component rapidly migrates to the uterine cavity, with a parallel increase in myometrial thickness, allowing complete and safe myoma excision during second-step hysteroscopy (Donnez *et al*., 1990; Bettocchi *et al*., 2004; Stamatellos and Bontis, 2007; Tan and Lethaby, 2013; Di Spiezio Sardo *et al*., 2008, 2015).

With all the techniques described here, there is risk of fluid (glycine) absorption while using monopolar energy. This risk is avoided by use of bipolar or laser energy with saline solution.

Hysteroscopic myomectomy is effective for control of bleeding, but failures are reported and are often related to growth of fibroids in other sites, association of fibroids with adenomyosis, and incomplete treatment of large intramural (partially submucous) myomas (Pritts, 2001; Pritts *et al*., 2009; Donnez and Jadoul, 2002; Donnez *et al*., 2014a; Parazzini *et al*., 2015).

In terms of reproductive outcomes, most studies are retrospective ([Bibr dmw023C21][Bibr dmw023C20],b). They report post-surgery pregnancy rates ranging from 16.7% to 76.9%, with a mean of 45% (Donnez *et al*., 2014a). Their robustness could be criticized (Metwally *et al*., 2011, 2012), but the authors of a recent review (Bosteels *et al*., 2015) acknowledge that the benefits of hysteroscopic removal of submucous myomas for improving the chances of pregnancy ‘cannot be excluded’. In addition, one prospective randomized study (Casini *et al*., 2006) has provided good-quality evidence that surgical therapy (hysteroscopic myomectomy) yields higher pregnancy rates than alternative treatments in women with submucous myomas.

### Laparoscopic myomectomy

Laparoscopic myomectomy is perceived by many gynecological surgeons to be more difficult, but the advantages are real: less severe post-operative morbidity, faster recovery with laparoscopic procedures and no significant difference between reproductive outcomes after laparoscopic or abdominal myomectomy (by minilaparotomy) (Donnez *et al*., 2014a,b; Bhave Chittawar *et al*., 2014; Segars *et al*., 2014). However, there have been reports of uterine rupture after laparoscopic myomectomy, thus emphasizing the importance of adequate closure of the myometrial defect (Dubuisson *et al*., 2000; Parker *et al*., 2010; Thomas *et al*., 2010). In a review of nine trials including 808 patients (Bhave Chittawar *et al*., 2014), there was no evidence of any difference in recurrence risk between laparoscopy and open myomectomy.

Usually, a 10 mm scope and two or three ancillary ports are used, although some gynecologists prefer a 5-mm scope. Depending on the site of the myoma, either a vertical (longitudinal) or a transversal incision is made. In the majority of cases, a unipolar hook probe is utilized, but CO_2_ laser myomectomy is also performed in some departments. In certain cases, uterine artery ligation may be beneficial to reduce intraoperative bleeding (Hald *et al*., 2004; Liu *et al*., 2007; Alborzi *et al*., 2009; Thomas *et al*., 2010; Bae *et al*., 2011; Donnez *et al*., 2014a). Robotic laparoscopic myomectomy has been evaluated in a few retrospective series (Gargiulo *et al*., 2012; Pitter *et al*., 2013; Lewis *et al*., 2015) but no prospective study has been published yet and considerable scepticism remains about the real advantages of the technique (Carbonnel *et al*., 2014).

Leiomyomas are usually removed with a morcellator, although some gynecologists propose vaginal removal through the cul-de-sac of Douglas or minilaparotomy to avoid the risk of dispersing tissue fragments during sarcoma morcellation. The risk of uterine fragment dispersion with the subsequent appearance of pelvic adenomyotic masses and parasitic leiomyomas was described in 2006 (Donnez *et al*., 2006, 2007) and remains a concern. This complication can be avoided by extensive peritoneal lavage and careful removal of all the fragments (Donnez *et al*., 2007), even if some authors still have misgivings (Pereira *et al*., 2015). Indeed, since their first publication, Donnez *et al*. no longer encountered this complication in a subsequent series of 400 laparoscopic hysterectomies (LHs), when caution was exercised and attention was paid to examine all areas of the abdominal cavity by placing the patient in the Trendelenburg and anti-Trendelenburg position and by extensive lavage (Donnez and Donnez, 2010). The risk of morcellation of uterine leiomyosarcomas has recently become a ‘hot’ topic, since the Food and Drug Administration (FDA) warned about the use of electromechanical power morcellation (Ton *et al*., 2015; Parker *et al*., 2015, 2016). It should nevertheless be stressed that the prevalence of sarcoma in leiomyomas is <0.3% and the debate on the use of electric morcellation has probably been overstated, not only because of the fear of medicolegal issues but also due to emotional reasons (Donnez *et al*., 2014a,b; Parker *et al*., 2016). In a recent study (Bojahr *et al*., 2015), the prevalence of sarcoma was just 0.06% in a series of 10 731 uteri morcellated for myomas during LH. Of course, we should do all we can to improve the diagnosis of sarcoma, but a similar low incidence (1/2000) was observed in a very recent meta-analysis by Pritts *et al*., (2015) and in a retrospective study including 4791 women in Norway (Lieng *et al*., 2015). Another meta-analysis by Brohl *et al*. (2015) concluded that leiomyosarcomas are diagnosed unexpectedly after surgery for what are presumed to be benign fibroids in 1 in 340 women, and that risks increase with age from less than one case per 500 women aged under 30 years to 1 in 98 women aged 75–79 years.

The technique of power morcellation in a bag was recently suggested to minimize the risk of inadvertent tissue spread (Kanade *et al*., 2014; Kho and Brown, 2015; Cholkeri-Singh and Miller, 2015), but there is no evidence that this technique will not increase the rate of post-operative complications (Donnez *et al*., 2014a,b).

Contraindications to laparoscopic myomectomy usually include the presence of an intramural myoma >10–12 cm in size or multiple myomas (≥4) in different sites of the uterus, requiring numerous incisions.

The dimensions and localization of the main myoma are the principal criteria for choosing the laparoscopic approach (Dubuisson *et al*., 2000; Alessandri *et al*., 2006; Palomba *et al*., 2007; Nezhat *et al*., 2009; Malzoni *et al*., 2010; Thomas *et al*., 2010; Donnez *et al*., 2014a,b; Segars *et al*., 2014; Parazzini *et al*., 2015). Thus, depending on the skill of the surgeon and his/her ability to suture the myometrial defect without delay, either laparoscopy or minilaparotomy may be selected.

In terms of infertility, several non-controlled studies have suggested that myomectomy yields a decrease in the miscarriage rate in women with myomas distorting the uterine cavity (Saravelos *et al*., 2011; Bernardi *et al*., 2014; Parazzini *et al*., 2015).In a review of prospective and retrospective studies, Donnez and Jadoul reported a pooled pregnancy rate of 49% (95% CI 46–52) in patients who underwent laparoscopic myomectomy (Donnez and Jadoul, 2002). In another review by Somigliana *et al*. (2007), the post-operative pregnancy rate was 57%. These post-myomectomy pregnancy rates have been confirmed by other studies, but the lack of randomized trials represents a serious drawback (Galliano *et al*., 2015). However, it should be pointed out that there are no significant differences in cumulative pregnancy rates or obstetric or perinatal outcomes when laparoscopic and abdominal myomectomy are compared (Metwally *et al*., 2011; Fukuda *et al*., 2013; Shen *et al*., 2015; Tian *et al*., 2015).

### Laparoscopic hysterectomy

Hysterectomy has long been considered standard surgical treatment for symptomatic intramural and submucous fibroids, particularly for women not wishing to conceive or those of premenopausal age (40–50 years). In the US, more than 600 000 hysterectomies are performed each year (Flynn *et al*., 2006). In Denmark, the overall hysterectomy rate was around 180/100 000 women during the period 1977–2011 (Lykke *et al*., 2013).

Fibroids are the main indication for hysterectomy and, in the last decade, LH has become the ideal surgical approach to replace laparotomy. In some departments, the rate of LH exceeds 90% (Donnez *et al*., 2009). Vaginal hysterectomy (VH) still remains indicated in some conditions, depending on the skill and habits of the surgeon (Aarts *et al*., 2015).

Some studies have reported an increased risk of complications after LH (Johnson *et al*., 2005; Aarts *et al*., 2015), but in a very large series, Donnez *et al*., (2009) found a similar complication rate after LH, VH and abdominal hysterectomy (0.44% of major complications). In a personal prospective series of 400 cases, no major complications were encountered (Donnez and Donnez, 2010). Of course, as stressed by the authors, uterine volume of ≥13–14 weeks represents a relative contraindication. A very recent study demonstrated that in some conditions, hospitalization for LH could be less than 5 h (Donnez *et al*., 2015c).

Some ‘in bag’ morcellation techniques, one of them called the Sydney technique, were developed to address the concerns of morcellating large myomatous uteri after total or subtotal hysterectomy (Mc Kenna *et al*., 2014) but, as stressed earlier, no large studies have demonstrated any real benefit in terms of general safety. Moreover, the risk of leiomyosarcoma morcellation during LH must be weighed against procedure-related complications associated with laparotomy, including mortality (Siedhoff *et al*., 2015).

### Laparoscopic cryomyolysis and thermo-coagulation

Both laparoscopic cryomyolysis and thermo-coagulation have the same goal: reduction or suppression of the primary blood supply and induction of myoma shrinkage by causing sclerohyaline degeneration (by very low or very high temperatures).

For cryomyolysis, a cryoprobe is inserted into the myoma and cooled to a temperature of <90°C (Zupi *et al*., 2004; Exacoustos *et al*., 2005). For laparoscopic thermocoagulation, either a monopolar or bipolar probe is inserted into the myoma before delivering the electrical current. In some studies, laser fibers (YAG) have also been used (Donnez *et al*., 2000). The limitation of all of these techniques is the lack of histological evaluation of the fibroids (Zupi *et al*., 2015).

### Laparoscopic occlusion of the uterine arteries

Laparoscopic occlusion of the uterine arteries appears to have no specific advantage over vaginal occlusion, as it requires a laparoscopic approach. Moreover, when compared to UAE, the outcomes were found to be inferior in terms of myoma size reduction and devascularization (Hald *et al*., 2004).

## Alternatives to surgical intervention

The alternatives to surgical interventions are illustrated on the right-hand side of Fig. 4.

### Uterine artery embolization

Although rapidly adopted by enthusiasts, the introduction of UAE has varied widely across the globe (Moss and Christies, 2016). This technique was first used in 1995 (Ravina *et al*., 1995) to treat uterine fibroids in women wishing to preserve their uterus.

UAE constitutes complete uterine therapy, as most fibroids are supplied by the uterine arteries. In UAE, percutaneous ablation of the fibromatous uterus is applied to induce ischemic necrosis of the fibroids, while the myometrium revascularizes. Most fibroids are targeted simultaneously. UAE has been shown, in randomized trials, to result in QoL similar to that achieved after surgery, but with a shorter hospital stay and earlier resumption of normal activities (Gupta *et al*., 2012, 2014).

Although UAE is highly effective for treating symptoms (reduction in bleeding and fibroid size), the risk of reoperation is a reality: 15–20% after successful embolization and up to 50% in cases of incomplete infarction (Kroencke *et al*., 2010; Gupta *et al*., 2014; Mara and Kubinova, 2014; Vilos *et al*., 2015, Spies, 2016). Among possible complications, abdominal pain due to ischemic necrosis of fibroids and risk of infection should not be overlooked (Goodwin and Spies, 2009). The impact of UAE on the ovarian reserve is another concern (Gupta *et al*., 2014), but a systematic review of 15 randomized trials and prospective cohort studies demonstrated that loss of ovarian function occurred primarily in women over 45 years of age (Kaump *et al*., 2013). In a very recent review, Zupi *et al*. (2015) clearly detailed the results and complications of UAE. It was emphasized that a desire for future pregnancy is a relative contraindication, as the lack of data in the literature cannot ensure a good pregnancy outcome. In a randomized controlled trial (RCT) comparing UAE and myomectomy, surgical removal had a more favorable outcome than UAE in terms of pregnancy rate (78% vs 50%), delivery rate (48% vs 19%), and abortion rate (23% vs 64%) (Mara *et al*., 2008).

Gupta's article in the Cochrane Database of Systematic Reviews (Gupta *et al*., 2014) evaluated seven RCTs comparing UAE and surgery (abdominal hysterectomy or myomectomy). The authors clearly stated that there were limitations in the evidence. The main limitations of the studies were a serious lack of precision due to wide confidence intervals, failure to clearly report methods, and the absence of blinding for subjective outcomes. They also estimated that between 15% and 32% of subjects would require further surgery within two years of UAE.

Several trials comparing UAE and myomectomy and UAE with focused ultrasound are currently ongoing, whose outcomes are awaited (Stewart, 2015).

### High-frequency magnetic resonance-guided focused ultrasound surgery

High frequency magnetic resonance-guided focused ultrasound surgery (MRgFUS) is thermal ablation using MRI to visualize the myoma and define the target. Ultrasonic energy is directed to a point inside the fibroid and coagulation tissue necrosis is induced in the myoma. In theory, damage to surrounding tissue is minimal (Clark *et al*., 2014; Park *et al*., 2014) but, in fact, the impact on critical neighboring structures cannot be excluded (Fischer *et al*., 2015; Kim *et al*., 2015).

As stressed by Zupi *et al*. in their review (2015), hyperintensive MRI images are associated with reduced treatment success compared with hypointensive images of fibroids. The principal limitations to the use of MRgFUS are that (i) only a fraction of patients with fibroids meet the inclusion criteria, (ii) future fertility may be compromised and (iii) the financial burden may be too heavy.

The literature is still scarce on this topic and additional studies are needed to evaluate safety profiles (Zupi *et al*., 2015). In a recent study, 30% of women underwent further fibroid surgery or procedures two years after MRgFUS (Jacoby *et al*., 2015). Screening and MRI-based prediction models for assessing therapeutic responses may reduce the risk of treatment failure (Kim *et al*., 2016). Several non-controlled clinical trials (Rabinovici *et al*., 2010; Berman *et al*., 2015) have reported pregnancies after MRgFUS, but a recent review by Clark *et al*. (2014) found a high rate of complications in the 34 documented pregnancies.

### Vaginal occlusion of the uterine arteries

Occlusion of the uterine arteries with a clamp-like device that remains in place for 6 h leads to myoma ischemia by interfering with the blood supply to the uterus (Hald *et al*., 2004). However, this technique is not recommended for women wishing to conceive in the future.

In a study by Vilos *et al*. (2006), dominant fibroid volume decreased by 24% and heavy bleeding symptoms decreased by 51%. Here too, research with larger populations is needed to prove the efficacy of the technique.

## Why we need new options

Fibroids are highly prevalent and represent a high health burden. Indeed, about 30% of women with leiomyomas will request treatment due to morbidities such as heavy menstrual bleeding, abdominal pain, pressure symptoms and/or infertility. Current treatments are mainly surgical and expensive. Among 600 000 hysterectomies performed each year in the USA, 200 000 are for fibroids (Flynn *et al*., 2006). In a study by Flynn *et al*. (2006), health care costs for the management of leiomyomas were estimated to be over $2 billion per year. There is no doubt that fibroids have a significant economic impact (Cardozo *et al*., 2012; Soliman *et al*., 2015), but the cost of therapy both to the health care system and women with fibroids must be balanced against the cost of untreated disease conditions, as well as the cost of ongoing or repeated investigations and treatment modalities (Vilos *et al*., 2015). Despite the lack of relevant medico-economic evaluations of the different therapeutics, it is likely that reducing the number of hysterectomies and other surgical procedures will reduce costs and morbidity. It is therefore necessary to develop and evaluate alternatives to surgical procedures especially when fertility preservation is the goal ([Bibr dmw023C63][Bibr dmw023C49],b).

## Current medical therapy

Two recent Cochrane Reviews on the use of herbal preparations (Liu *et al*., 2013) and aromatase inhibitors (Song *et al*., 2013) concluded that there was no evidence to support the use of herbal preparations or aromatase inhibitors as medical therapy for treating myomas. However, as reviewed by Islam *et al*. (2013, 2014), there is evidence to suggest that certain dietary or alternative treatments like phytochemical herbal preparations may be effective. In addition, some synthetic and natural compounds as well as growth factors are now under laboratory investigation (Islam *et al*., 2013), while observational data suggest that increased consumption of fruits, vegetables and low-fat dietary products are associated with a reduced risk of developing fibroids (Wise *et al*., 2011). Nevertheless, some uncertainty remains due to insufficient high-quality studies with large enough sample sizes.

### GnRH agonists

**Figure 5:**
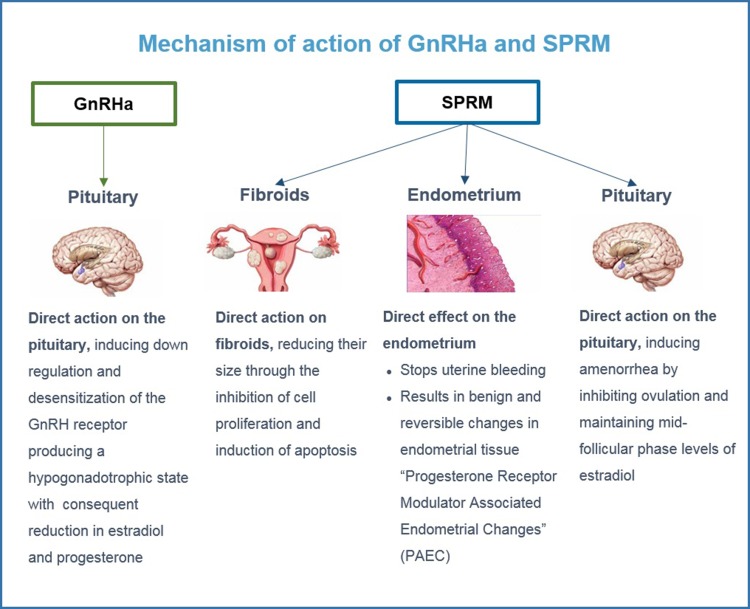
Mode of action of GnRH agonists and SPRMs (Selective Progesterone Receptor Modulators). GnRH agonists have a direct impact on the pituitary. SPRMs have a direct impact on fibroids, endometrium and the pituitary.

By inducing a state of hypoestrogenism and temporary menopause with amenorrhea, GnRH agonists have been used to shrink fibroids and restore hemoglobin levels in symptomatic women (Donnez *et al*., 1989, 1990; Carr *et al*., 1993; Lethaby *et al*., 2001) (Fig. 5). They cannot be used for long periods of time because of their side effects, such as hot flushes and bone loss. A very recent review demonstrated that there is modest evidence that add-back therapy (tibolone, raloxifene, estriol and ipriflavone) can help reduce bone loss and that medroxyprogesterone acetate (MPA) and tibolone may moderate vasomotor symptoms (Moroni *et al*., 2015).

Use of GnRH agonist before surgery is still a matter of debate, but a review by Gutmann and Corson (Gutmann and Corson, 2005) reports that ‘preoperative use of GnRH agonist appears to be relevant and beneficial in patients with submucous fibroids’. Benefits include a resolution of preoperative anemia (Donnez *et al*., 1989; Lethaby *et al*., 2001; Stamatellos and Bontis, 2007; Doherty *et al*., 2014); a decrease in fibroid size (Donnez *et al*., 1989; Lethaby *et al*., 2001); a reduction of endometrial thickness and vascularization with subsequently improved visibility and reduced fluid absorption (Donnez and Jadoul, 2002; Metwally *et al*., 2011; Doherty *et al*., 2014) and the possibility of surgical scheduling (Donnez *et al*., 1990; Donnez and Jadoul, 2002; Pritts, 2001; Pritts *et al*., 2009). Conversely, this preoperative treatment is associated with post-injection endometrial bleeding due to the flare-up effect.

## The future of medical therapy

### Evidence of the crucial role of progesterone pathways in the pathophysiology of uterine fibroids by use of selective progesterone receptor modulators

To date, genetic and epigenetic factors, sex steroids, growth factors, cytokines, chemokines and ECM components have been identified as being implicated in the pathogenesis of leiomyomas (Bulun, 2013; Islam *et al*., 2013; Marsh *et al*., 2015; Protic *et al*., 2015; Yin *et al*., 2015). Many growth factors and also activin and myostatin play a role in the mechanisms involved in the development of leiomyomas (Ciarmela *et al*., 2011a,b; Protic *et al*., 2015). Of course, estrogen and progesterone and their respective receptors also have a very significant impact on leiomyoma growth (Kim and Sefton, 2012). Recently, Wong *et al*. (2016) demonstrated that testosterone was additionally implicated in the growth of uterine fibroids. *In vitro* studies have also shown that fibroid development depends on miRNA regulation of gene targets which impact cellular processes (Karmon *et al*., 2014). The initial event that triggers the first stages of tumorigenesis nevertheless involves somatic mutations (Kim and Sefton, 2012).

In the past, estrogen was considered to be the major growth factor in myoma development. However, already in the 1990s, a number of studies reported increased the expression of both progesterone receptor A (PR-A) and progesterone receptor B (PR-B) in leiomyoma tissue (Englund *et al*., 1998; Nisolle *et al*., 1999) compared with adjacent normal myometrium. Very recently, Tsigkou *et al*. showed that PR-B mRNA and PR-A and PR-B proteins were more concentrated in leiomyomas than in matched myometrium (Tsigkou *et al*., 2015). Levels of PR-B mRNA in leiomyoma tissue were directly associated with the number of myomas, but inversely correlated with the intensity of symptoms. Moreover, higher proliferative activity, demonstrated by proliferating cell nuclear antigen (PCNA) and the mitotic index, was observed in leiomyomas during the luteal (secretory) phase (Nisolle *et al*., 1999). There is evidence from preclinical and clinical trials, as well as from histological and pharmacological studies, that progesterone and its receptors play a key role in uterine fibroid growth (Bouchard *et al*., 2011; Bouchard, 2014; Chabbert-Buffet *et al*., 2011, 2012, 2014; Kim and Sefton, 2012; Bestel and Donnez, 2014; Moravek *et al*., 2015). In a review, Kim and Sefton (2012) described, in detail, the activation of signaling pathways in leiomyomas by both estrogen and progesterone. Progesterone is able to cause rapid, membrane-initiated effects, independent of gene transcription, that alter the production of second messengers involved in cell signaling transduction pathways. The PI3K/AKT pathway is mediated by progesterone which, through its receptors, can quickly activate this pathway, which is increasingly considered to be a potential promoter of leiomyoma growth. PTEN, on the other hand, should be considered as a negative regulator of AKT (Kim and Sefton, 2012). Progesterone and growth factor signaling pathways are interconnected and govern numerous physiological processes such as proliferation, apoptosis and differentiation.

Progesterone can modulate the expression of growth factor signaling proteins and is implicated in the regulation of genes associated with proliferation and apoptosis, but these genes have not yet been fully identified or studied in detail (Islam *et al*., 2013; Kim and Sefton, 2012; Moravek *et al*., 2015). There is therefore evidence that progesterone plays a crucial role, but the mechanism by which it promotes proliferation, the repertoire of genes involved, and how it crosstalks with growth factor signaling pathways all need to be investigated in greater depth. The recent discovery of stem cells and their paracrine interactions with more differentiated cell populations within leiomyoma tissue may lead to the development of therapeutics that temper leiomyoma growth as well as those that eradicate them (Moravek *et al*., 2015).

Having established the crucial role of progesterone in the growth and development of myomas, we can modulate the progesterone pathway by use of selective progesterone receptor modulators (SPRMs) (Chabbert-Buffet *et al*, 2005, 2011, 2015; Bouchard *et al*. 2011; Bouchard, 2014; Kim and Sefton, 2012; Bestel and *Donnez* 2014; Donnez *et al*., 2012a,b). SPRMs are synthetic compounds that exert either an agonistic or antagonistic effect on PRs (Fig. 5). Their binding allows these receptors to interact with coactivators and/or corepressors, and this is further impacted by the presence of coregulators in a particular cell type, which will dictate whether an SPRM acts more as an agonist or antagonist (Chabbert-Buffet *et al*., 2005, 2011). Hence, the mechanism of action of SPRMs on PRs depends on their structure and how they alter the PR conformation, resulting in exposure or inactivation of particular binding domains. Their activity is also mitigated by tissue types and physiological contexts (Kim and Sefton, 2012; Bouchard, 2014; Moravek *et al*., 2015).

### SPRMs and fibroids: what we know so far

Four members of the family of compound SPRMs have been investigated in phase II clinical trials: mifepristone, asoprisnil, ulipristal acetate (UPA) and telapristone acetate (Spitz, 2009; Bouchard *et al*., 2011; Bouchard, 2014; Chabbert-Buffet *et al*., 2011; Nieman *et al*., 2011; Shen *et al*., 2013; Whitaker *et al*., 2014). All were shown to decrease leiomyoma size and reduce uterine bleeding in a dose-dependent manner. However, although three studies (Fiscella *et al*., 2006; Engman *et al*., 2009; Bagaria *et al*., 2009) showed a myoma volume reduction of ±30%, a review of the literature by Tristan *et al*. (2012) (Cochrane Review) found no clear evidence of this. Some follow-up studies have also raised concerns about unopposed estrogenic activity and liver toxicity (Williams *et al*., 2007; Spitz, 2009; Bouchard *et al*., 2011; Chabbert-Buffet *et al*., 2011; Tristan *et al*., 2012).

The latest antiprogestin to be studied in large clinical trials, UPA, has shown promising results in terms of efficiency and safety. UPA was compared to a placebo and to leuprolide acetate (a GnRH agonist) in two randomized trials (Donnez *et al*., 2012a,b). In these first clinical studies, uterine bleeding was controlled in more than 90% of patients receiving a three-month course of UPA, and the median times to control bleeding were shorter in the UPA group (5–7 days) than in the GnRH agonist group (21 days). The control of bleeding and subsequent correction of anemia were clinically relevant (Barlow *et al*., 2014; Donnez *et al*., 2012a,b). Indeed, it has been well documented that preoperative anemia, even to a mild degree, is associated with an increased risk of morbidity and mortality in patients undergoing surgery (Mussalam *et al*., 2011; Richards *et al*., 2015). UPA was also found to have a sustained effect (up to six months) in women who did not undergo surgery after the three-month study period. By contrast, those treated with GnRH agonist experienced rapid regrowth of their fibroids, whose size reached pre-therapy dimensions by six months post-treatment (Donnez *et al*., 2012a,b).

Importantly, the induced effects of SPRMs on the endometrium, now described as progesterone receptor modulator (PRM)-associated endometrial changes (PAECs) (Mutter *et al*., 2008) (Fig. 5), present in almost 70% of patients at the end of treatment, have proved to be benign and reversible, as they disappeared two months after the end of therapy (Williams *et al*., 2012; Donnez *et al*., 2012a,b). Safety has also been well documented in pharmacokinetic studies following multiple doses (Pohl *et al*., 2013, 2015).

The mechanism of action by which SPRMs reduce menstrual blood loss in women with fibroids remains unknown (Wilkens *et al*., 2013), although a number of possible factors have been proposed by Williams *et al*. (2007, 2012). Wilkens *et al*. (2013) reported that uterine NK cells regulate endometrial bleeding and were suppressed by asoprisnil.

### Long-term intermittent administration of SPRMs, opening up new treatment perspectives

Because of the sustained effect observed in the first two trials (Donnez *et al*., 2012a,b), additional intermittent (12-week) courses of SPRMs with off-treatment intervals may be an alternative for long-term medical management of fibroids. The results of the first long-term intermittent administration study suggested that more than one course of SPRMs can maximize its potential benefits in terms of bleeding control and fibroid volume reduction (Donnez *et al*., 2014b).

**Figure 6:**
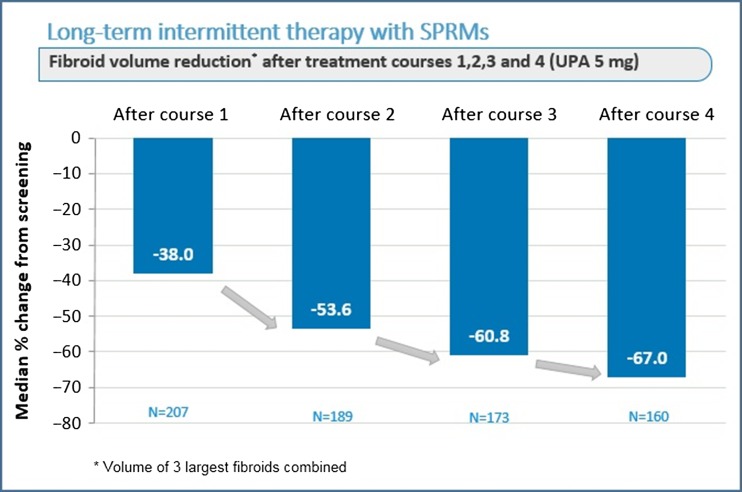
Effect on fibroid volume reduction after four courses of three months of ulipristal acetate (UPA) 5 mg daily. The off-period between two courses was two natural cycles.

Adapted from Donnez *et al*. (2015a, 2016).

The latest clinical trial was initiated to investigate the efficacy and safety of four repeated 12-week courses of either 5 or 10 mg UPA daily for intermittent treatment of symptomatic uterine fibroids ([Bibr dmw023C50][Bibr dmw023C53],b,c). This study demonstrated a similar degree of response in both treatment groups. We will therefore focus on the results in terms of efficacy and safety of this trial using the approved dose of 5 mg UPA in a repeated intermittent therapy setting (four courses) ([Bibr dmw023C50][Bibr dmw023C47],c). The percentages of subjects identified as being in amenorrhea after individual treatment courses (1, 2, 3 and 4 in the study) were 75.8%, 84.1%, 86.4% and 87.5% in the 5 mg group ([Bibr dmw023C50][Bibr dmw023C53],c). The pictorial blood assessment chart (PBAC) (Higham *et al*., 1990) score was measured at initial screening and after 1, 2 and 4 courses to assess the level of menstrual bleeding during the off-treatment period. In the 5 mg group, (median) levels at screening were 224.0, dropping significantly with each subsequent course and finally reaching 77.5 after course 4 ([Bibr dmw023C50][Bibr dmw023C53], c). The percentage of subjects with a clinically significant volume reduction of ≥25% increased from course 1 to course 4 (from 62.3% to 78.1%), and those with a volume reduction of ≥50% also increased from course 1 to course 4, proving that repeated courses considerably maximize the impact of treatment. This was also proved by the volume reduction of the three largest fibroids which was increased from course 1 to course 4 (Fig. 6). The findings of this study therefore demonstrate the efficacy of 5 mg UPA treatment and further confirm the safety of repeated intermittent administration of UPA for symptomatic myomas ([Bibr dmw023C50][Bibr dmw023C53],b,c).

The safety profile of UPA during multiple treatment courses was well documented in this study ([Bibr dmw023C50][Bibr dmw023C53],b,c). Safety assessments, including vital signs, physical examinations and laboratory analyses, as well as reported adverse events (AEs) both on and off treatment, showed repeated intermittent administration of UPA to be well tolerated. The vast majority of AEs (97.6%) were of mild or moderate severity. Headaches and hot flushes were the most frequently reported AEs (less than 11% of subjects in any treatment course), but the frequency of these events decreased with each additional treatment course. Breast pain or discomfort was observed in 3% of subjects. In this series of 451 women (Donnez *et al*., 2015b, c), serious AEs related to medication included five cases of menorrhagia, one bipolar disorder, one spontaneous myoma expulsion, one abdominal pain and one back pain. No safety concerns were identified from physical examination, vital signs, ovarian ultrasound or electrocardiogram (ECG).

Based on the available data related to endometrial safety after up to four treatment courses, no increased occurrence of more serious conditions of the endometrium, such as hyperplasia with atypia or endometrial carcinoma, was noted. The frequency of SPRM-associated non-physiological endometrial changes (PAEC) did not appear to increase with repeated treatment courses, reaching 13.3% after a fourth treatment course, and returning to pretreatment levels within three months of completion of treatment. These data further confirm the rapid reversibility of PAEC following completion of treatment and subsequent menstruation. It is reassuring that median endometrial thickness (7–8 mm) was similar to screening levels after single and multiple treatment courses and remained stable during post-treatment follow-up (three months after treatment cessation).

A recent study by Courtoy *et al*. suggested an important role of UPA in collagen degradation induced by matrix metalloproteinase 2 (MMP-2), offering an explanation for the sustained beneficial effect. Indeed, this study strongly points to multifactorial mechanisms of action involving: (1) a persistently low cell death rate; (2) a limited period of cell death and (3) ECM remodeling concomitant with stimulation of MMP-2 expression (Courtoy *et al*., 2015).

An *in vitro* study demonstrated another possible mechanism of action of UPA: inhibition of activin A expression and function in cultured leiomyoma cells (Ciarmela *et al*., 2014).

### Novel approaches and algorithms, with a special emphasis on infertility

There is a clear need for alternatives to surgery, even the less invasive endoscopic techniques, especially when fertility preservation is the goal (Donnez *et al*., 2014a,b; Donnez *et al*. 2015b). There is no doubt that surgery remains indicated in some instances, but we must now establish whether SPRMs (UPA) allow less invasive surgery or even complete avoidance of surgery. On the other hand, it is clear that long-term intermittent use of UPA will change our approach to the management of uterine fibroids.

To address the question of which therapy to adopt, it is crucial to consider key factors determining the management of uterine fibroids: patient age, severity of symptoms (pain, bleeding and infertility), wish to preserve the uterus and/or fertility, localization of fibroids according to FIGO classification and myoma volume. The approaches described below are according to the FIGO classification (Munro *et al*., 2011).

#### Type 0 myomas

**Figure 7:**
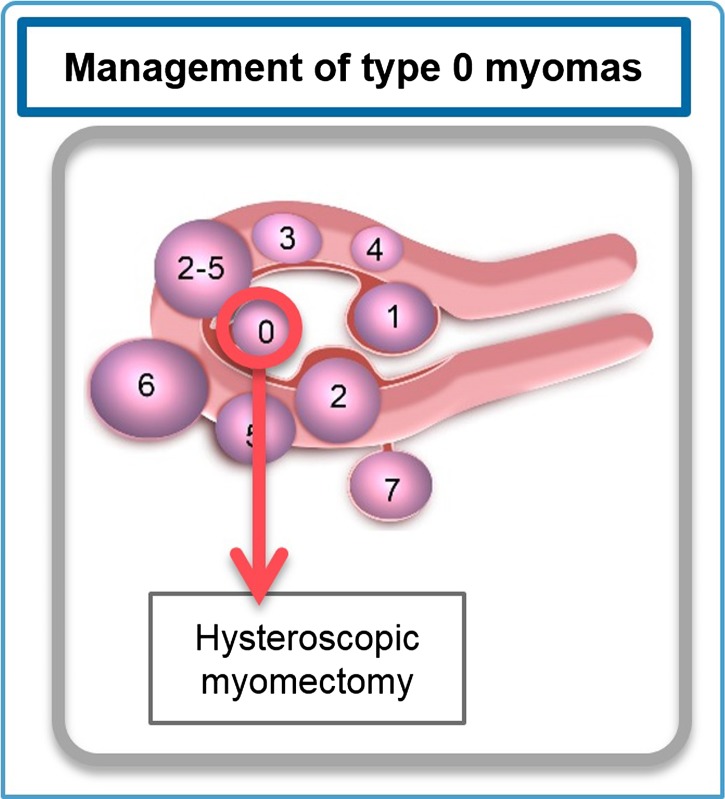
Management of type 0 myomas. Hysteroscopic myomectomy is the most appropriate approach. Fibroid classification cartoon republished with permission from Munro *et al*. (2011).

If type 0 myomas are present, cutting the pedicle by hysteroscopy is indicated (Fig. 7).

#### Type 1 myomas

**Figure 8:**
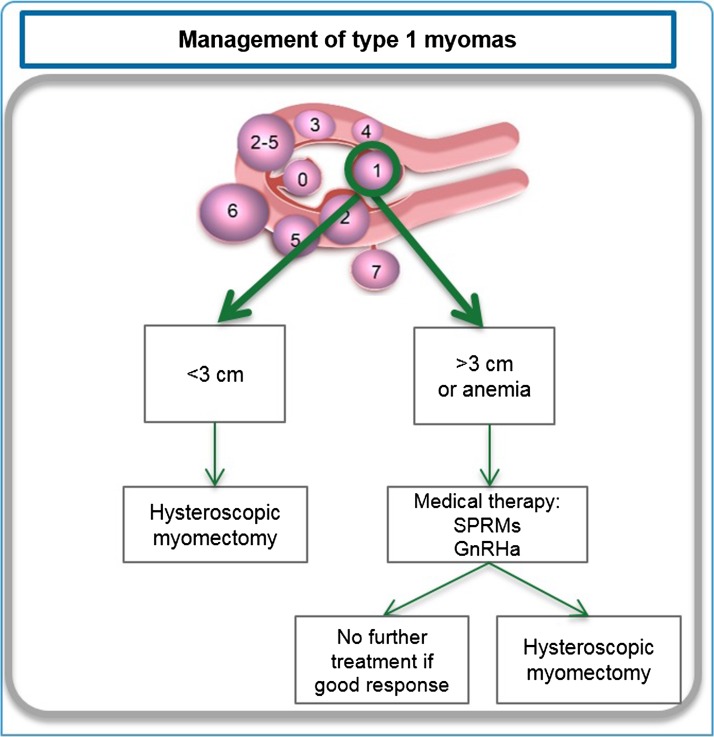
Management of type 1 myomas. Depending on the myoma size, presence of anemia and the surgeon's skill, hysteroscopic myomectomy combined or not with ulipristal acetate(UPA) should be proposed. Fibroid classification cartoon republished with permission from Munro *et al*.(2011).

In the majority of cases, hysteroscopic myomectomy for type 1 myomas is relatively straightforward for experienced surgeons, especially in case of type 1 myomas less than 3 cm in size (Fig. 8). If a fibroid is of type 1 but larger than 3 cm, or if the patient presents with anemia, pre-hysteroscopic medical therapy (SPRMs or GnRH agonist) is indicated. Results in terms of subsequent fertility after hysteroscopic myomectomy were discussed earlier in this paper.

Medical therapy may be given in one or two courses of three months. In the vast majority of cases, type 1 myomas respond to this preoperative therapy and regress in size, enabling an easier hysteroscopic approach in better conditions (recovery of hemoglobin). It should be pointed out that in some cases, myomas regress so much that surgery may be avoided.

#### Type 2 or type 2–5 myomas (single or multiple) distorting the uterine cavity

##### Young infertile women of reproductive age and wishing to conceive

**Figure 9:**
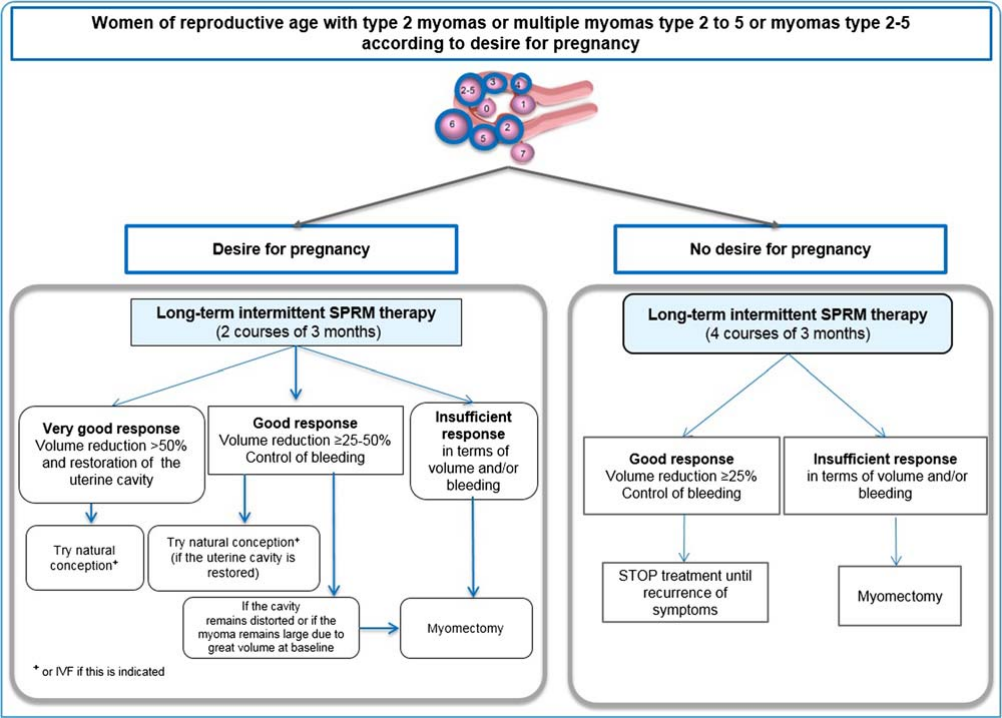
Management in case of myomas or multiple myomas (type 2–5) in women of reproductive age, according to desire for pregnancy. In cases of infertility, two courses of three months are recommended (left panel). Subsequent therapy is determined depending on the response to treatment and restoration of the uterine cavity. If there is no desire to conceive (right panel), long–term (four courses) intermittent therapy may be proposed. In case of a good response in terms of fibroid volume reduction and bleeding, treatment is stopped and only restarted if symptoms recur. Fibroid classification cartoon republished with permission from Munro *et al*. (2011).

In case of type 2 myomas, medical therapy (SPRMs) can be proposed (Fig. 9). Myomas often respond to this preoperative therapy and regress in size. This reduction also allows a hysteroscopic approach that can be planned after the first menstrual bleed (Donnez *et al*., 2014a,b). In some cases (if myomas regress so much that they no longer distort the uterine cavity), surgery may not be required. If myomas are multiple (≥2) or of different types (type 2–5), as is frequently observed, medical therapy (SPRMs) can be given in two courses of three months, as described in clinical trials with UPA (Donnez *et al*., 2014a,b; [Bibr dmw023C50][Bibr dmw023C53],b). After these two courses of three months, there are three possible outcomes.

**Figure 10:**
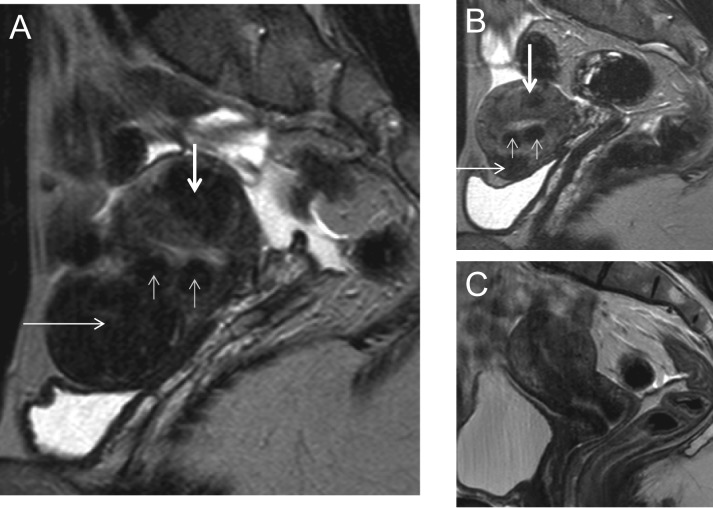
Considerable shrinkage of all myomas after four courses of intermittent ulipristal acetate (UPA) therapy. A patient aged 30 years presented with heavy menstrual bleedingand an unclear desire for pregnancy. **(A)** Before treatment, a midline sagittal T2-weighted magnetic resonance image (MRI) demonstrated the presence of multiple myomas: type 2, 3, 4 and 6. **(B)** Upon completion of treatment (intermittent UPA therapy (four courses of threemonths), the uterine cavity was no longer distorted. **(C)** One year after delivery of a healthy baby, no fibroid regrowth was observed after delivery.

The most positive outcome would be that myoma regression is very significant (>50% decrease in volume). The uterine cavity is no longer distorted and the patient can try to conceive naturally or undergo assisted reproductive techniques, if indicated. A first series of pregnancies after UPA treatment was recently described, demonstrating that in some cases, surgical treatment is not required and patients can conceive and deliver healthy offspring (Luyckx *et al*., 2014) (Fig. 10). Other case reports have also been published (Monleon *et al*., 2014). In our series of pregnancies, patients were able to have unprotected sexual intercours or to start with ovarian stimulation after the second menstrual bleed (Luyckx *et al*., 2014). For those having to undergo IVF, a vaginal ultrasound was performed on day 3 of the second menstrual bleed to assess the absence of a thick endometrium. The second outcome would be that myoma regression is significant (≥25% but <50%). However, in some instances, if the uterine cavity remains distorted or if the myoma remains large due to great volume at baseline, the indication for surgery stands. In this case, medical treatment may allow surgery to be performed by a laparoscopic approach once the hemoglobin level is normalized, avoiding laparotomy.

The least outcome would be that the response to medical therapy is inadequate. In this case, surgery remains indicated.

##### Young women of reproductive age with symptomatic myomas and wishing to preserve their fertility but having no immediate desire for pregnancy

**Figure 11:**
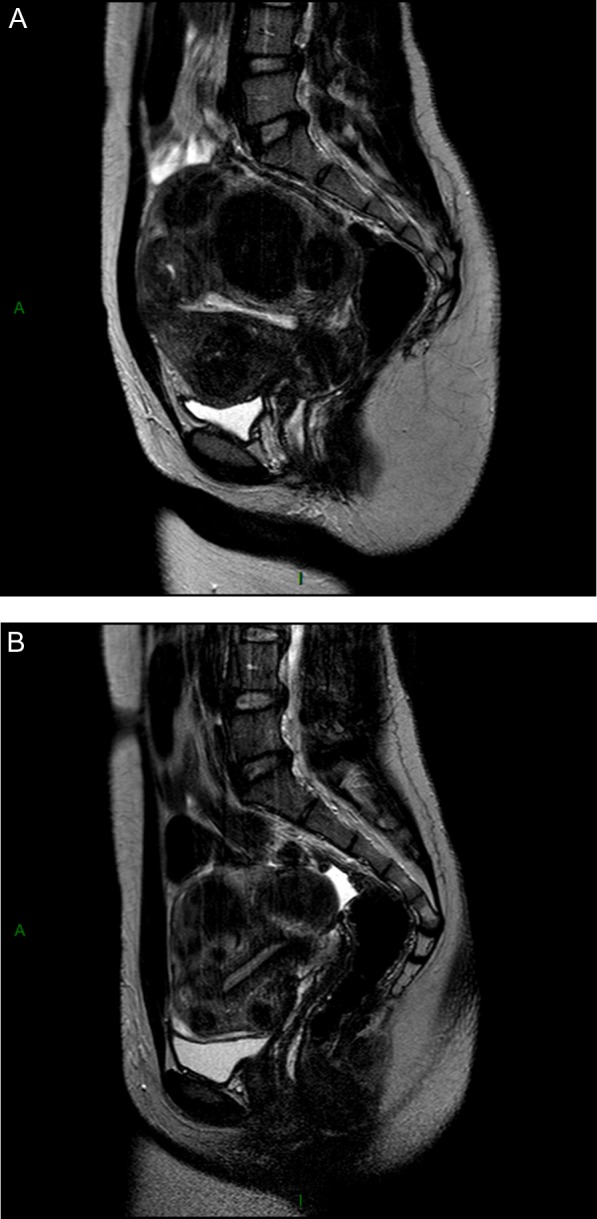
Important shrinkage of the submucosal myoma was obtained after two courses of three months of intermittent ulipristal acetate (UPA) therapy. **(A)** Coronal T2-weighted magnetic resonance image (MRI) image illustrated the presence of multiple myomas (type 2, type 2-5) distorting the uterine cavity in a 19-year-old nulligravid patient, who presented to the emergency department, with heavy menstrual bleeding and anaemia (haemoglobin level of 7.4 g/l).The patient received two courses of UPA (5 mg) and iron. **(B)** At the end of therapy, MRI demonstrated a significant reduction in myoma volume (<50%) and restoration of the uterine cavity. Amenorrhea was achieved, with a haemoglobin level of 11.9 g/l. The patient was free of symptoms and did not wish to conceive; therefore, surgery was avoided.

In these cases too, medical therapy can be proposed (Fig. 9), taking into account recent clinical trials with SPRMs demonstrating that four courses of three months induce a significant improvement, course upon course (decrease in myoma size and PBAC score) ([Bibr dmw023C50][Bibr dmw023C53],b, 2016). In the vast majority of cases, regression of myoma size (≥25% in 80% of patients) and control of bleeding (in >90% of patients) will allow avoidance of surgery and restoration of hemoglobin levels (Fig. 11).

When there is no immediate wish to conceive, there is no pressing need for surgery (even if the uterine cavity remains distorted and/or large myomas are still present). In some cases, myomas will all but disappear. In case of symptom recurrence, medical therapy may be reinitiated. Myomectomy should only be considered when the patient wishes to become pregnant, and if really necessary according to the localization and volume of the fibroids still present. This is important to take into account, especially for women of African descent. Indeed, African and African-American women have a greater chance of developing symptomatic myomas at an earlier age than Caucasian women (Baird *et al*., 2003). It is widely known that the rate of recurrence of myomas after myomectomy can reach almost 60% after an interval of 4–5 years, and that the risk of pelvic adhesions is significantly increased after a repeated myomectomy (Malone, 1969; Donnez *et al*., 2014a). Medical treatment with SPRMs can thus be beneficial, since long-term intermittent therapy (repeated in case of symptom recurrence during the interval) may help to avoid or at least postpone the need for surgery until the patient wishes to conceive (Fig. 11).

Surgery therefore remains indicated only when the patient wishes to conceive, and if large myomas (>3–4 cm) distorting the uterine cavity are present, as these could be the cause of her infertility.

##### Asymptomatic women with myomas and undergoing IVF or oocyte donation

A meta-analysis by Pritts *et al*. (2009) evaluating 23 studies showed a significant drop in pregnancy and implantation rates in the presence of myomas, especially submucous and/or intramural myomas distorting the uterine cavity. In another meta-analysis, Sunkara *et al*. (2010) demonstrated their impact on fertility, even in case of intramural myomas not distorting the uterine cavity. A recent study by Yan *et al*. (2014) confirmed that intramural fibroids >2.85 cm in size significantly decreased the delivery rate of patients undergoing IVF/intracytoplasmic sperm injection (ICSI).

Moreover, some centers have large cohorts of patients of more than 40 years of age in oocyte donation programs (Cobo *et al*., 2015). In this group of women, the prevalence of myomas is higher than in women of 30 years of age.

It could be proposed that patients with myomas be treated with one or two courses of SPRMs before IVF or oocyte donation, in order to reduce the size of myomas and restore the uterine cavity and subsequently improve implantation rates. Clinical trials evaluating UPA before IVF or oocyte donation should be initiated to investigate this further.

##### Premenopausal women presenting with symptomatic myomas and with no desire for pregnancy but a wish to keep their uterus

**Figure 12:**
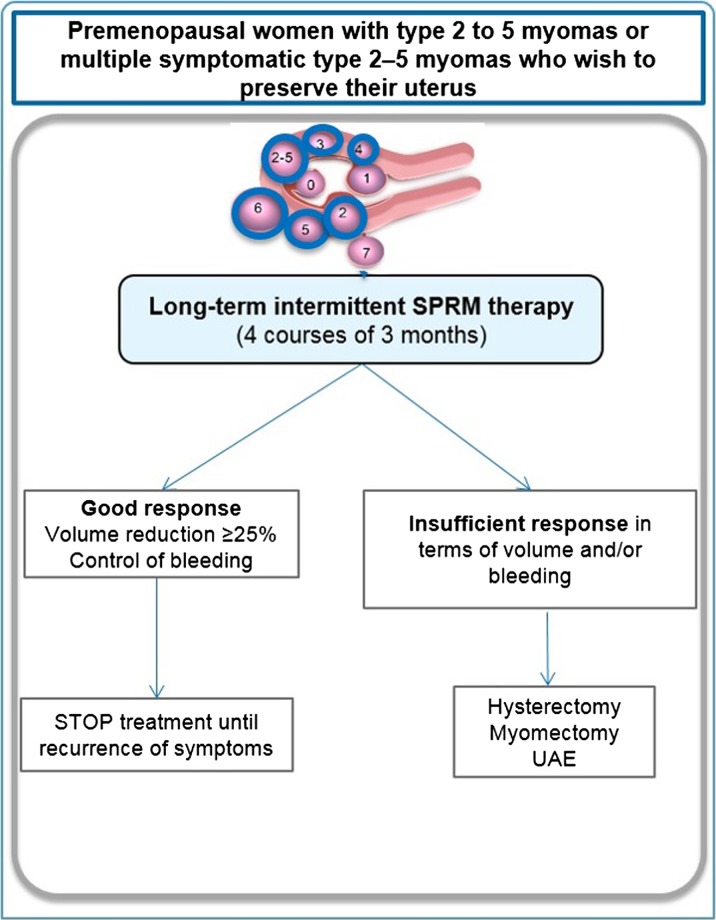
Management of type 2 to 5 myomas or multiple myomas (type 2–5) in premenopausal women wishing to preserve their uterus. In this case, long-term (four courses of three months) intermittent therapy with SPRMs is proposed. Fibroid classification cartoon republished with permission from Munro *et al*. (2011).

Isolated type 2 fibroids are relatively rare in premenopausal women. In the majority of cases, patients with symptomatic myomas have an enlarged uterus with multiple myomas or large myomas of type 2–5 (Fig. 12).

Our latest results ([Bibr dmw023C50][Bibr dmw023C53],b, 2016) led us to slightly modify previously published algorithms (Donnez *et al*., 2014a,b) for this group of women. Indeed, in subjects treated with 5 mg UPA for four courses of three months, the percentage of patients with a clinically significant volume reduction increased from 62.3% after 1 course to 78.1% after 4 courses, suggesting increased benefits with repeated courses. The percentage of women showing a clinically significant reduction of >50% also increased from course 1 (37.2%) to course 4 (63.8%). Moreover, the median PBAC score during the off-treatment period decreased with each subsequent course.

In case of a good response (characterized by a clinically significant volume reduction and/or control of bleeding), treatment can be stopped after four courses and the patient is re-evaluated ([Bibr dmw023C50][Bibr dmw023C53],b, 2016). Repeated therapy may be proposed when the symptoms recur, as no endometrial hyperplasia was diagnosed in subjects who took 5 mg UPA for eight courses of three months. In this context, the goal is to reach menopause without the need for surgery. Data indicating that SPRMs exert an anti-proliferative effect in breast tissue are also reassuring (Poole *et al*., 2006; Engman *et al*., 2008). Some studies reported anti-proliferative effects on the endometrium after SPRM courses of up to six months (Wilkens *et al*., 2009).

### Uterine fibroid associated pathologies

Endometriosis and adenomyosis are frequently associated with uterine fibroids (Donnez *et al*., 2014a).

#### Endometriosis

In theory, induction of amenorrhea in women treated with SPRMs should also relieve endometriosis-associated pain. In mammalian models, SPRMs stop prostaglandin production by endometriotic lesions (Gemzeel-Danielson and Hamberg, 1994; Elger *et al*., 2004) and this direct effect may also serve to reduce pain.

**Figure 13:**
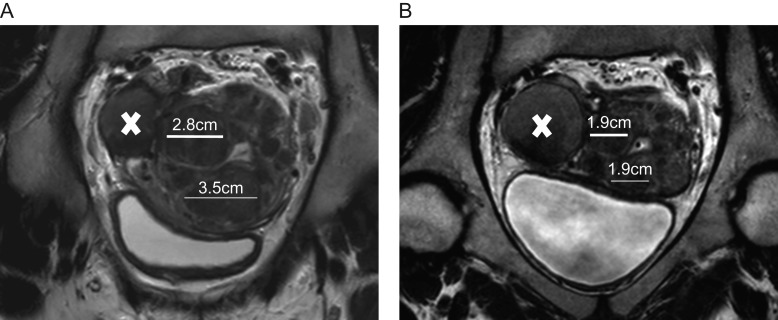
27 year-old women complaining of heavy menstrual bleeding and pelvic pain. A: Coronal T2-weighted MRI images illustrated the presence of type 2–5 and type 3 myomas distorting the uterine cavity and an endometrioma (indicated by X) of 4.3 cm in size. The white lines represent the diameter of the myomas. This patient received long-term intermittent therapy with 5 mg of UPA (2 courses of 3 months). B: At the end of therapy there was an important reduction in myoma volume, but not endometrioma volume.

Nevertheless, it should be stressed that even if endometriosis and uterine fibroids are both estrogen-dependent diseases, they show a completely different response to progesterone: endometriosis is characterized by progesterone resistance (Donnez *et al*., 1996; Nisolle and Donnez, 1997; Bulun, 2009), while fibroids grow under the influence of progesterone (Kim and Sefton, 2012). Fig. 13 shows the excellent response (>50% volume reduction) to UPA obtained in fibroids, but the absence of response (or even a slight volume increase) in endometriomas.

The specific effects of SPRMs have yet to be determined in ectopic endometrium. Indeed, PAECs (Williams *et al*., 2007, 2012; Mutter *et al*., 2008) may be present in lesions, as observed in eutopic endometrium.

#### Adenomyosis

Adenomyomas and adenomyosis are two distinct clinical entities. Adenomyomas may respond very well to SPRMs, but severe full-thickness adenomyosis, characterized by the presence of numerous sites of ectopic endometrium in the myometrium of an enlarged uterus, is a specific entity that might have a completely different response.

SPRMs will probably be effective in reducing adenomyosis-associated pain by inducing amenorrhea, but are unlikely to be able to significantly reduce the size of the uterus. Clinical trials are ongoing to explore this particular context and the impact of endometrial modifications, as PAECs will also be present in ectopic intramyometrial endometrium.

### Future prospectives for medical therapy

**Figure 14:**
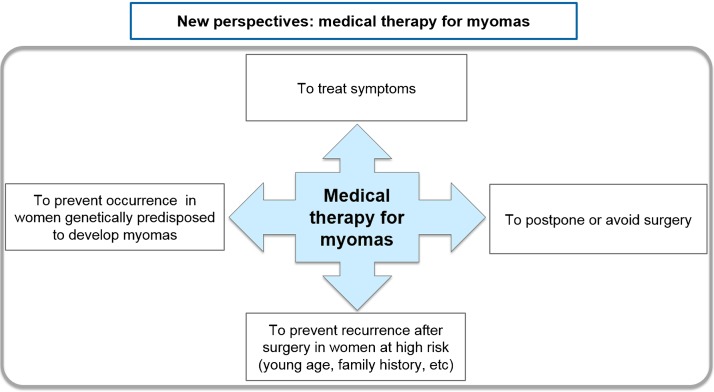
New avenues are emerging in medical fibroid therapy. The first goal of medical therapy is clearly to treat symptoms resulting from the presence of fibroids (heavy menstrual bleeding, pelvic pain, bulk symptoms, infertility, etc.), as well as to postpone or avoid surgery. Further avenues should be investigated by randomized trials, looking to avoid recurrence after surgery in women at high risk of recurrence, and to prevent occurrence of myomas in genetically predisposed women.

SPRMs have opened up new avenues to explore in fibroid medical therapy, to both treat symptoms and postpone or to eliminate the need for surgery. Future clinical trials should focus on prevention strategies, such as preventing occurrence in women genetically predisposed to this condition, and avoiding recurrence after surgery in women at high risk (i.e. those of a young age or with a family history) (Fig. 14).

## Conclusion

Symptomatic uterine fibroids require surgical and/or medical therapy according to the severity of symptoms, age, infertility, wish to preserve the uterus and FIGO classification (Fig. 14). Current strategies involve mainly surgical intervention, such as hysterectomy, myomectomy by hysteroscopy and myomectomy by laparoscopy or laparotomy. Hysterectomy provides the most effective treatment for fibroids, but is not appropriate in many cases. The choice between less invasive techniques (uterine-sparing options such as myomectomy) is guided by the size, number and location of fibroids as well as the personal experience of the gynecologist and available equipment. Other surgical techniques, such as laparoscopic cryomyolysis, thermocoagulation or uterine artery occlusion, are rarely used. Non-surgical interventions, such as UAE and MRgFUS, are also available but the desire for future pregnancy is a relative contraindication.

On the other hand, the need for medical therapy remains a reality. It is indeed essential that new treatments be developed to be able to offer as there is a pressing need for alternatives to surgical intervention, particularly when fertility preservation is the goal.

GnRH agonists have been used to shrink fibroids and restore hemoglobin levels in symptomatic women, but because of their side effects, they cannot be used for long periods of time. However, there is now growing evidence of the crucial role of progesterone in pathways in the pathophysiology of uterine fibroids by the use of SPRMs. UPA (one member of the SPRM compound family) has been studied in large clinical trials and its long-term intermittent administration has been evaluated, yielding promising results for new treatment perspectives. It was found that more than one three-month course of UPA maximizes its potential benefits in terms of bleeding control and fibroid volume reduction. Hence, depending on age and symptoms (infertility, bleeding, etc.), SPRMs should be considered an alternative to surgical therapy, or at least an adjunct to surgery, in some circumstances, as illustrated in the algorithms.

**Figure 15:**
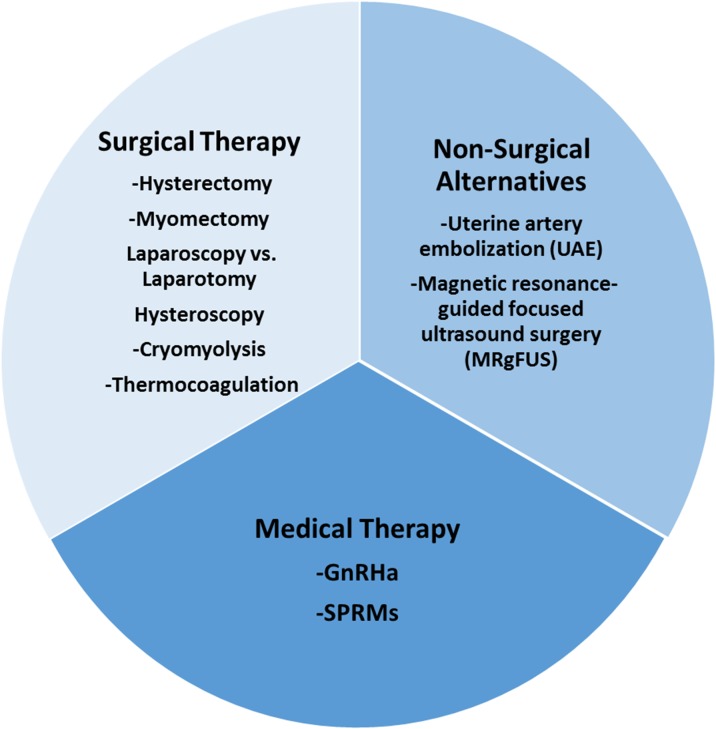
Surgical, non-surgical and medical therapy for the management of fibroids: the current armamentarium.

In conclusion, asymptomatic fibroids do not require treatment once the diagnosis is confirmed by ultrasonography or MRI. Women should be made aware of all available treatment options (medical, radiological and surgical) and why they may or may not be appropriate. Gynecologists now have new tools in their armamentarium (Fig. 15) opening up novel strategies for the management of uterine fibroids.
